# Testing the link between visual suppression and intelligence

**DOI:** 10.1371/journal.pone.0200151

**Published:** 2018-07-06

**Authors:** Sandra Arranz-Paraíso, Ignacio Serrano-Pedraza

**Affiliations:** 1 Faculty of Psychology, Universidad Complutense de Madrid, Madrid, Spain; 2 Institute of Neuroscience, Newcastle University, Newcastle upon Tyne, United Kingdom; University of Waterloo, CANADA

## Abstract

The impairment to discriminate the motion direction of a large high contrast stimulus or to detect a stimulus surrounded by another one is called visual suppression and is the result of the normal function of our visual inhibitory mechanisms. Recently, Melnick et al. (2013), using a motion discrimination task, showed that intelligence strongly correlates with visual suppression (*r* = 0.71). Cook et al. (2016) also showed a strong link between contrast surround suppression and IQ (*r* = 0.87), this time using a contrast matching task. Our aim is to test this link using two different visual suppression tasks: a motion discrimination task and a contrast detection task. Fifty volunteers took part in the experiments. Using Bayesian staircases, we measured duration thresholds in the motion experiment and contrast thresholds in the spatial experiment. Although we found a much weaker effect, our results from the motion experiment still replicate previous results supporting the link between motion surround suppression and IQ (*r* = 0.43). However, our results from the spatial experiment do not support the link between contrast surround suppression and IQ (*r* = -0.09). Methodological differences between this study and previous studies which could explain these discrepancies are discussed.

## Introduction

In a recent study, Melnick et al., [1] have shown a strong link between intelligence and visual surround suppression. In particular, in a direction discrimination task, participants with high IQ showed low duration thresholds for small moving stimuli and high duration thresholds for large moving stimuli (duration threshold is defined as the minimum time needed to discriminate the correct direction of motion). These authors computed a Surround Suppression Index (SSI) subtracting both duration thresholds in logarithmic units [2] and found a significant positive correlation between IQ and SSI (*r* = 0.71). The basic idea underlying this link is that the suppression index carries information about two aspects: our ability to suppress irrelevant visual information and also how fast we process relevant visual information. These aspects are closely related to IQ (for a deeper discussion linking this low-level psychophysical measurement and IQ, see [3]). However, the recent evidence fails to replicate this finding ([4]; *r* = -0.01). Troche et al., [4] couldn’t rule out the possibility that stimulus and apparatus differences between both studies could be behind this failure to replicate the aforementioned finding. They suggested that the link between IQ and motion surround suppression is probably confined to a specific range of stimulus parameters. In a different study, using a contrast matching task, Cook et al. [5] also found a stronger correlation between contrast surround suppression and visuospatial IQ (*r* = 0.87). In this case, the suppression of irrelevant information and the effect of the GABAergic inhibition could explain the link between IQ and contrast surround suppression.

In the present study, our main objective is to test this link using two psychophysical tasks that provide a measurement of visual surround suppression. We will use a similar motion discrimination task, used previously by Melnick et al. [1] and a contrast detection task [6–8].

### Motion discrimination task

Previous psychophysical results have shown that the time needed to discriminate the correct direction of motion (duration threshold) depends on the contrast, the size, and the speed of a given stimulus [9, 10] (see a review in [11] and in [3]). The most interesting result shows that, at high contrasts, duration thresholds increase with increasing stimulus size [9, 12–15]. This surprising result has been explained by the operation of a suppressive center-surround mechanism [3, 9]. This mechanism has been linked to neural surround suppression, in particular, to the existence of neurons with a center-surround antagonism that are located in the middle temporal area (MT) [9, 16–19]. Those neurons show a response pattern that is consistent with the psychophysical results; that is, their firing rate is reduced for large stimuli presented at high contrasts [20] and brief durations [21].

The strength of the psychophysical suppression has been quantified by means of a Motion Suppression Index (MSI) defined as MSI = log_10_(θ_big_)−log_10_(θ_small_), where θ_big_ and θ_small_ are the duration thresholds for the big and small moving stimuli respectively (high MSI values indicate a strong suppression) [2].

The presumed link between the psychophysical results and the operation of a center-surround inhibitory neural mechanism has been used to indirectly estimate the neural strength of the suppressive center-surround interactions in special populations (see a review in [3]). The hypothesis is that a stronger cortical inhibition causes an improvement in motion direction discrimination for large stimuli at high contrasts. That is to say, it indirectly causes a low surround suppression index. In recent years, many studies have shown evidence that supports this hypothesis in different populations; for example, older adults [22–25], young children [26] and patients with schizophrenia [2], depression [27], epilepsy [28], or autism [29]. However, recent studies have not replicated the results found previously in autism [30] and depression [31].

Many of these studies have linked the reduced psychophysical surround suppression to a dysfunction of GABA-ergic inhibitory cortical function ([2, 22, 24, 25, 27]). This link is based on evidence that suggests that aging, schizophrenia, and depression are associated with GABA-ergic alterations ([32–35]). However, there is recent data that does not support this link. For example, in primates, Liu & Pack [36] showed that manipulations of GABA levels in MT had no effect on surround suppression. In particular, they found that local blockade of GABA receptors did not diminish surround suppression. Liu et al. [37] have recently found that the injection of GABA or local manipulations of the efficacy of the GABAergic inhibition had little influence on surround suppression. Schallmo et al. [38], combining psychophysics and magnetic resonance spectroscopy, have found that suppression in humans is not primarily driven by GABAergic inhibition. Another example by Read el al., [39] showed that acute alcohol intoxication had no effect on SSI. This result is surprising provided that alcohol affects GABAergic inhibition in many cortical areas [40] and that low alcohol concentrations enhance the inhibition of the GABAergic system [41]. Therefore, one would expect an increment in the suppression strength under the effects of alcohol, which is not the case.

### Contrast detection task

It is well known that the contrast thresholds for detecting a target grating increase if that target is surrounded by a grating with the same spatial frequency and orientation [6, 7, 42–47]. However, when the surround is orthogonally oriented there is an improvement in contrast detection under some conditions; this is, contrast thresholds for the target presented with the surrounding grating are lower than those for the target without the surround [7, 48]. These effects of the surround are attributed to the spatial surround suppression processing that takes place in V1 [49]. Evidence coming from physiology studies in cats, macaques, and mice have shown that, in some conditions, a strong surround suppression can be obtained when a visual neuron is stimulated outside of its classical receptive field [50–55]. As it happens in the case of psychophysics, surround suppression is stronger when the stimulus presented in the surround and the center of the neuron’s receptive field has the same spatial frequency and orientation.

Contrast surround suppression also affects the apparent contrast of a target depending on the contrast of the surround stimulus. For example, if the contrast of the surround is higher than that of the target, then the apparent contrast of the target is reduced [44, 45, 56–59].

GABA is the main neurotransmitter underlying cortical inhibition and, on the other hand, its concentration becomes reduced by 10% in schizophrenia patients [33]. For this reason, different studies have tested the effect of contrast suppression in patients with schizophrenia by measuring apparent contrast [33, 60] and contrast detection thresholds [48]. In all these studies, the authors found a reduced surround suppression.

Recently, Cook et al. [5] used an apparent contrast matching task with first and second order gratings in a center-surround configuration [61]. They found a strong and significant positive correlation between cortical GABA levels and contrast surround suppression (*r* = 0.88). This result confirms and extends previous results by Yoon et al. [33] in schizophrenia patients and controls (r = 0.76). In Cook et al’s study, the authors also found a significant correlation between visual cortical GABA levels and visual intelligence (*r* = 0.83) and between contrast surround suppression and intelligence (*r* = 0.87).

In this study, we test the link between IQ and visual surround suppression using a similar motion discrimination task to the one previously used by Melnick et al. [1], and we also test, for the first time, the link between IQ and contrast surround suppression using a contrast detection task [6–8].

## Materials and methods

### Participants

Fifty volunteers, 16 males and 34 females ranging from 18 to 28 years old (mean ±SD, 20.74 ± 2.44 years) who were unaware of the purpose of the study took part in the experiments. Those 50 participants were the final sample after applying two exclusion criteria: a) to be older than 30 years and younger than 18 years and b) to have an abnormal vision. We included the aging criterion because aging influences surround suppression. In particular, motion surround suppression decreases with aging [22, 23]. We also tested visual acuity (for both eyes and two distances 40 cm and 300 cm) and 3D vision using the ETDRS 2000 series visual acuity chart and the Frisby Stereotest, respectively. All participants had normal or corrected-to-normal vision. Only participants with visual acuity lower than logMAR = 0.5 (in both eyes) and participants with stereovision (stereoacuity < than 500 arcsec) took part in the experiments. For each participant, we measured his/her depression level using the Beck Depression Inventory (BDI-II, 0.85 of internal-consistency reliability). This was done because motion surround suppression has been seen to be affected in patients with depression [27, 31]. The mean ± SD of the BDI results was 10.7 ± 9.3. This score is considered a low level of Depression (Note: no correlation was found between the suppression indices of both experiments and BDI results: motion experiment *r* = 0.09, *p* = 0.605; spatial experiment, *r* = 0.008, *p* = 0.96). All participants provided written informed consent and took part in the experiments voluntarily. Finally, the experimental procedures were approved by the Complutense University of Madrid Ethics Committee (Faculty of Psychology), and the study complies with the Code of Ethics of the World Medical Association (Declaration of Helsinki).

### Apparatus

We used the same equipment for both the motion and the spatial experiments. All stimuli were presented on a gamma-corrected 17-in Eizo Flex Scan T565 CRT monitor under the control of a Mac Pro 3.7 GHz Quad-Core Intel Xeon E5 (graphics card AMD FirePro D300 2048 MB) running Matlab (The MathWorks, Inc, Natick, MA) using the Psychophysics Toolbox extensions [62–64] with 14-bits of gray-scale resolution (DataPixx Lite, VPixx Technologies Inc., Canada, http://www.vpixx.com). The luminance was corrected using a Minolta LS-110 photometer (Konica Minolta Optics, Inc., Osaka, Japan). The monitor had a resolution of 800 × 600 pixels (horizontal × vertical) with a vertical frame rate of 148 Hz, mean luminance of 49.1 cd/m^2^, and was observed binocularly from a distance of 55 cm in a dark room. A chin rest (UHCOTech HeadSpot, Houston, TX) was used to stabilize the head of the participants and to control the observation distance. Responses were recorded using the ResponsePixx Handheld (VPixx Technologies Inc., Canada).

### Stimuli

**Fig 1:**
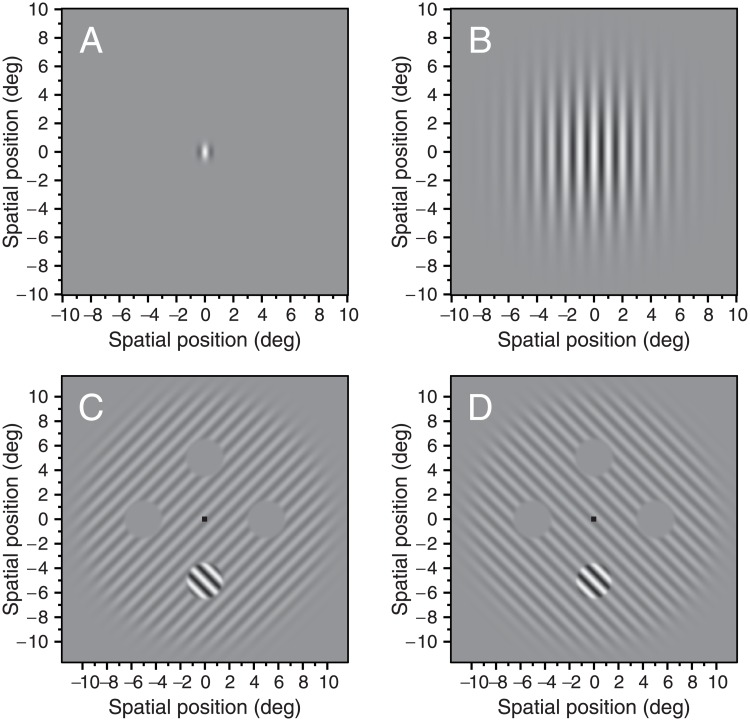
Examples of the stimuli used in the motion and spatial experiments. (A-B) stimuli used in the motion experiment. (C-D) stimuli used in the spatial experiment. **A)** Gabor patch of 1 c/deg, 92% contrast, and a diameter of 0.7 deg (diameter = 2 × σ_xy_). **B)** Gabor patch of 1 c/deg, 92% contrast, and a diameter of 6 deg. **C)** Example of the orthogonal surround condition. Target of 1 c/deg surrounded by a grating with the same spatial frequency; **D)** Example of the parallel surround condition. The contrast of the surround was fixed to 25%. On each trial, the target appeared randomly in one out of four possible positions.

All stimuli were created in Matlab (The MathWorks, Inc, Natick, MA). In the *motion experiment* (direction discrimination task), the stimuli were vertical Gabor patches of 512 × 512 pixels with 8-bits luminance range, presented in the center of the monitor in a square of 19.5 × 19.5 cm subtending a visual angle of 20.1 × 20.1 deg. The remainder of the screen was set at the mean luminance. The Gabor patches had a Michelson contrast of 92%, a spatial frequency of 1 c/deg and drifted rightwards or leftwards at a speed of 2 deg/sec. We measured duration thresholds for two different diameters: 0.7 and 6 deg (diameter = 2 × σ_xy_; where σ_xy_ is the standard deviation in x and y of the spatial Gaussian window in units of degrees of visual angle) (see Fig 1A and 1B). The contrast of the Gabor patch was modulated using a temporal Gaussian envelope given by, *m*(*t*) = *M*exp{−*t*^2^ / (2*σ*_*t*_^2^)}, where *M* is the peak contrast (92%). We defined the duration of the stimuli as twice the temporal standard deviation (2 × *σ*_t_) [9]. The overall duration of the presentation interval was 1000 msec. The participants were instructed to maintain fixation on a small cross (0.25 × 0.25 deg) presented in the center of the screen before the presentation of the stimuli. The luminance of the small cross was also modulated with a Gaussian temporal envelope with a standard temporal deviation of 80 msec. The cross disappeared before the presentation of the stimulus.

For the *spatial experiment* (contrast detection task) we designed the stimuli based on previous research [6–8, 48]. The target was a sinusoidal grating of 1 c/deg windowed with a 10^th^-order Butterworth spatial window of 3 deg diameter (see [65], p.170). The target was located at 5 deg eccentricity in one out of four possible positions. The surround was also a sinusoidal grating of the same spatial frequency with a fixed contrast of 25% windowed with a 20 deg 10^th^-order Butterworth spatial window (see Fig 1C and 1D). The surround had four “holes” of 3.05 deg, so between the target and surround there was a small gap of 0.05 deg. The orientation of the target changed randomly, taking values between ± 45° on every trial. Two conditions were tested, a) when the surround and the target were orthogonally oriented with respect to each other (Fig 1C); and b) when the surround and target had the same orientation (parallel) (Fig 1D). A third control condition, the target without any surround, was also tested. However, this condition was not used to compute the suppression index. In this case, the phase of the surround and the target was the same but changed randomly adopting values between 0 and 2π on every trial. The contrast of the stimulus was modulated by a temporal Gaussian envelope with a fixed temporal standard deviation of 100 msec.

### Procedure

Before starting the experiments, we measured the visual acuity, the stereoacuity, and we administered a depression test (see Participants section). Only the subjects that met our inclusion criteria were selected to participate in the experiments. Then, each participant performed both of our psychophysical experiments and the IQ test in random order.

In order to measure the participant’s intelligence, we administered the Reynolds Intellectual Assessment Scales^™^ test (Spanish version) (RIAS test, [66, 67]). We measured general, verbal, and non-verbal intelligence (RIAS_general_, RIAS_verbal_, and RIAS_non-verbal_). These three IQ values are highly correlated with WAIS-III (RIAS_general_, *r* = 0.77; RIAS_verbal_, *r* = 0.63; RIAS_non-verbal_, *r* = 0.58; *p*<0.01; [68]). Administering this test takes about 40 min. We also administered the screening version for general intelligence, the Reynolds Intellectual Screening Test^™^ (RIST), that takes about 20 min (highly correlated with WAIS-III, *r* = 0.75, *p*<0.01; [68]).

In the *motion experiment* (motion discrimination task), the participants were instructed to fixate on a small cross presented on the center of the screen. Once the cross disappeared, a drifting Gabor patch appeared on the screen moving leftwards or rightwards randomly. The participant’s task was to indicate the direction of motion (left or right) by pressing a button. After the participant’s response, a new trial was initiated. The duration of the presentation (for details, see the Stimuli section) was controlled by a Bayesian adaptive staircase [69]. The particular characteristics of the staircase can be seen in Serrano-Pedraza et al. ([15] see the Procedure section). Duration thresholds, defined as the minimum presentation time of the drifting stimuli needed to discriminate the correct direction of motion, corresponded to twice the standard deviation of the temporal Gaussian envelope. Duration thresholds were defined as a stimulus presentation duration such that performance in a motion direction discrimination task was 82% correct responses. Each staircase stopped after 40 trials, where the mean of the final probability distribution corresponds to the value of the duration threshold ([70]). The staircases in each session were interleaved randomly for the small and large window sizes. In total, 12 duration thresholds were estimated: six thresholds per spatial window size (0.7 and 6 deg).

In the *spatial experiment* (contrast detection task) the participants were also instructed to fixate on a cross presented on the center of the screen; this time, the cross was a rotating one. The fixation cross was visible during the stimulus presentation in order to drive the participant’s attention to the center of the screen. The participant’s task was to identify the position where the target was presented. In order to measure the contrast detection threshold of the target, we used a spatial 4AFC task where the target randomly appeared in one out of four possible positions (see Fig 1C and 1D). The contrast of the target in each trial was controlled by a Bayesian adaptive staircase (see details in [48]). The contrast threshold was defined as the minimum contrast needed in order to detect the target with a performance of 62% correct responses. Each staircase stopped after 30 trials, and the mean of the final probability distribution was assumed as the value of the contrast threshold. The staircases were interleaved for the three conditions: parallel surround, orthogonal surround, and no surround. Three contrast thresholds were measured per condition.

In both the motion and the spatial experiments, no feedback about the correctness of the responses was provided and practice sessions were performed before starting.

### Suppression index

The strength of the psychophysical suppression in each experiment was quantified by means of a Motion Suppression Index (MSI) and a Contrast Suppression Index (CSI).

The suppression index MSI was defined as

$$\text{MSI}={log}_{10}({\text{D}}_{large})-{log}_{10}({\text{D}}_{small}),$$ (1)

where D_large_ and D_small_ are the duration thresholds, in msec, for the large and small moving stimulus respectively [2].

The suppression index CSI was defined as

$$\text{CSI}={log}_{10}({\text{C}}_{parallel})-{log}_{10}({\text{C}}_{orthogonal}),$$ (2)

where C_parallel_ and C_orthogonal_ are the Michelson contrast thresholds for the parallel surround (same orientation as the target) and the orthogonal surround respectively [8].

### Statistical analysis

The main objective of this study was to test the link between visual suppression and intelligence. In order to investigate this link, two different psychophysical tasks where each task provided an index of visual suppression (MSI and CSI) were used. Moreover, before starting with the statistical analysis, we determined the Cook’s distance in order to detect highly influential observations when regressing IQ and each visual suppression index. For the MSI score, we found three influential observations defined as participants with a Cook’s distance higher than three times the mean of all Cook’s distances. For the CSI suppression index, we found four influential observations using the same criterion. In both cases, the influential observations were not used in the analysis. Using a two-sided test, and because of our sample size (46–47), we could detect a significant correlation of *r* = 0.4, with β = 0.2 (power = 80%) and α = 0.05. Note that the correlations found previously were *r* = 0.71 [1] and *r* = 0.87 [5].

## Results and discussion

### Psychophysical experiments and IQ measurements

#### Motion experiment

**Fig 2:**
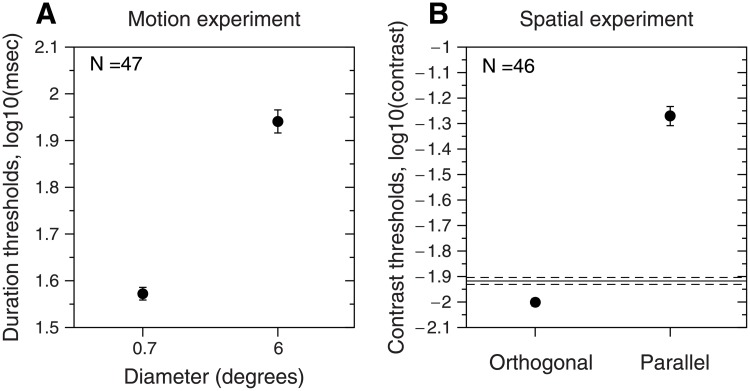
Results of motion and spatial experiments. **A)** Duration thresholds (2 × *σ*_t_) in logarithmic units (mean ± SEM) for each diameter tested (0.7 and 6 deg). **B)** Contrast thresholds (mean ± SEM) in logarithmic units for the orthogonal and parallel conditions. The horizontal line corresponds to the contrast thresholds (mean ± SEM) for the control condition without surround.

Fig 2 shows the results of both psychophysical experiments. Fig 2A shows the duration thresholds (log_10_(duration in msec)) for each window size (0.7 and 6 deg). These results replicate previous findings [1, 8, 9] where the duration thresholds for the small stimulus (mean ± SD was D_small_ = 1.57 ± 0.09 log_10_(msec), 38.2 ± 8.37 msec, N = 47) were significantly lower (repeated-measures t-test, t(46) = 17.37, *p* < 0.001, using logarithmic units, N = 47) than the duration thresholds for the large stimulus (mean ±SD was D_large_ = 1.94 ± 0.17 log_10_(msec), 94.06 ± 38.32 msec, N = 47). In particular, these results are similar to those obtained by Melnick et al., [1]: duration thresholds for small 39.13 ± 17.5 msec and large 80.25 ± 34.29 msec (N = 53) (window sizes 1.8 and 7.2 deg). However, these results are very different from those obtained by Troche et al., [4]: duration thresholds for small 82 ± 28 msec and large 136 ± 60 msec (N = 177) (window sizes 1.8 and 7.2 deg). Troche et al. [4] used a higher contrast than Melnick et al. [1] (95% vs. 42%, our study 92%), so higher duration thresholds for the large stimulus are to be expected. However, the duration thresholds for the small stimulus (1.8 deg) should have been much lower.

#### Spatial experiment

Fig 2B shows the contrast thresholds (in log units) for the three conditions: parallel surround (mean ± SD was C_parallel_ = -1.27 ± 0.25, N = 46), orthogonal surround (mean ± SD was C_orthogonal_ = -2.00 ± 0.10, N = 46), and no surround (mean ± SD was C_no-surround_ = -1.91 ± 0.1, N = 46). As expected, the condition with the highest contrast thresholds was the parallel surround one. These contrast thresholds are in agreement with previous results [6–8]. A repeated measures ANOVA with a Greenhouse-Geisser correction (ε = 0.604) demonstrated that the mean of the contrast thresholds, in log units, differed significantly between the three conditions (*F*(1.208,54.367) = 410,59, *p* < 0.001, η_p_^2^ = 0.90). Post hoc tests using paired t-tests with a Bonferroni correction showed significant differences between all conditions (*p* < 0.001).

#### Motion and contrast suppression indices

**Fig 3:**
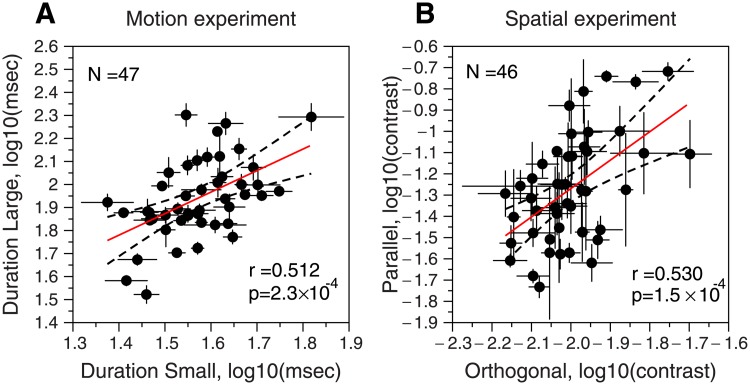
Scatter plots of motion and spatial experiments. **A)** Motion experiment results. Duration thresholds (in log values, mean ± SEM) for the large (D_large_) stimulus (6 deg) as a function of the duration thresholds for the small (D_small_) stimulus (0.7 deg). The red line shows the fitted regression line: D_large_ = 0.47 + 0.93 × D_small_. **B)** Spatial experiment results. Contrast thresholds (in log values, mean ± SEM) for the parallel condition (C_parallel_) as a function of the contrast thresholds for the orthogonal surround (C_orthogonal_). The red line shows the fitted regression line: C_parallel_ = 1.40 + 1.34 × C_orthogonal_. Pearson correlation (*r*) and *p* values are inserted in each panel. Dashed lines: 95% regression confidence interval.

Fig 3 shows the scatter plots for both experiments. Fig 3A shows the duration thresholds for the large stimulus (in log units) as a function of the duration thresholds for the small stimulus. These results show a significant positive correlation between the duration thresholds for the small and large stimulus (*r* = 0.51, *p* < 0.001, 95% Confidence Intervals CI = [0.26, 0.7], N = 47), which is in line with the correlation obtained by Melnick et al., [1] (*r* = 0.64, *p* < 0.001, N = 53).

The average Motion Suppression Index (MSI) obtained from our data was MSI = 0.37 ± 0.14 (mean ± SD), which is similar to the one in previous results: Yazdani et al. [8] (MSI = 0.40 ± 0.22, N = 36); Read et al. [39] (MSI = 0.31 ± 0.16, N = 56). This result is also similar to the MSI obtained by Melnick et al., [1] (MSI = 0.32 ± 0.15, N = 53), even though there are stimulus differences between both studies. For example, the speed we used was 2 deg/sec vs. 4 deg/sec, and our stimulus sizes were 0.7 and 6 deg vs. 1.8 and 7.2 deg. However, the MSIs found by Melnick et al. [1] and our study are much bigger than the MSI reported by Troche et al. [4] (MSI = 0.22 ± 0.16). Interestingly, the stimulus parameters used in Melnick et al., and Troche et al. are very similar; they used the same sizes (1.8 and 7.2 deg) and similar speeds 4.8 deg/sec (Troche et al.) vs. 4 deg/sec (Melnick et al.). The main difference between both studies was the contrast used: 42% (Melnick et al.) vs. 95% (Troche et al.). However, given that surround suppression is stronger at higher contrasts [9], it is hard to explain why Troche et al. [4] found a lower MSI value. These authors suggest that probably this small MSI is related to the different equipment used in their experiments [4].

Fig 3B shows the results of the spatial experiment. The panel represents the relationship between the contrast thresholds for the parallel-surround condition and the orthogonal-surround condition. Like in the motion experiment, the results show a significant positive correlation between the contrast thresholds for the orthogonal and parallel surround (*r* = 0.53, *p* < 0.001, 95% CI = [0.28, 0.71], N = 46). The average of the Contrast Suppression Index (CSI) obtained from this data was CSI = 0.73 ± 0.22 (N = 46) (mean ± SD), which is in agreement with the CSI previously reported by Yazdani et al. [8] (CSI = 0.56 ± 0.19, N = 36).

The Pearson correlation between MSI and CSI did not reach any statistical significance, *r* = 0.10, *p* = 0.47, 95% CI = [-0.18, 0.37], N = 50. This result replicates the main finding of Yazdani et al. [8], where they did not find any significant correlation between these two forms of psychophysical surround suppression (*r* = -0.19, *p* = 0.24, N = 36). Therefore, these results suggest that motion and contrast surround suppression reflect the activation of independent cortical mechanisms.

#### Intelligence results

With regard to the intelligence measurements, the mean ± SD of the IQ tests for the 50 participants were: RIAS_general_ = 106.2 ± 11.48 (range: 78–131); RIAS_verbal_ = 104.08 ± 13.17 (max = 126, min = 75); RIAS_non-verbal_ = 106.74 ± 10.12 (range: 82–133). The screening IQ test showed a mean ± SD of RIST_general_ = 104.38 ± 12.77 (range: 72–128). Although the means from RIST_general_ and RIAS_general_ were significantly different (repeated-measures t-test, t(49) = 2.53, *p* = 0.014), the IQ values from RIST_general_ and RIAS_general_ presented a strong positive correlation *r* = 0.91, *p*<0.001, 95%CI = [0.85, 0.95], N = 50. One must take into consideration, though, that the results from Melnick et al (2013) show higher values for general intelligence 112.92 ± 12.77 (mean ± SD; range: 75–147). We will discuss the possible effect of this difference in the Discussion section.

### Testing the link between IQ and Motion Suppression Index

**Fig 4:**
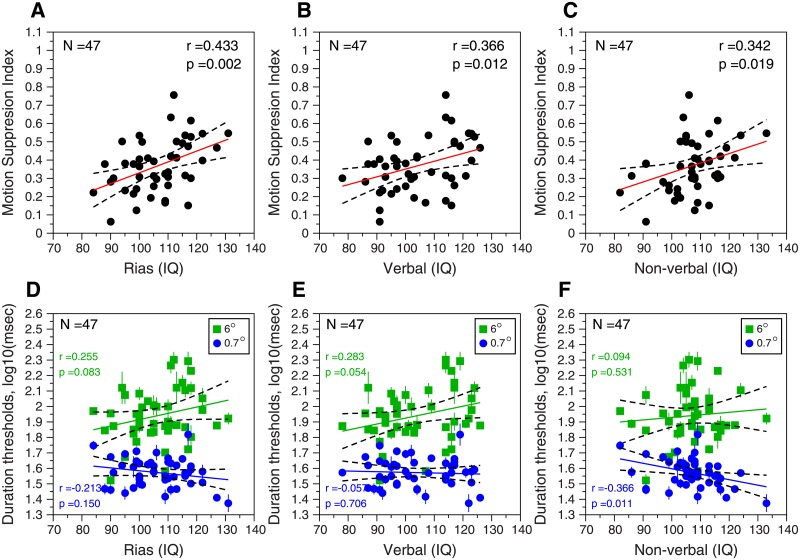
Relationship between Motion Suppression Index and IQ. **(A-C)** Motion suppression index (MSI) as a function of IQ, for general (A), verbal (B), and non-verbal (C) intelligence. The red lines represent the fitted regression line. General intelligence: MSI = -0.48 + 0.008 × IQ_g_; verbal intelligence: MSI = -0.26 + 0.006 × IQ_v_; and non-verbal intelligence: MSI = -0.25 + 0.006 × IQ_n-v_. **(D-F)** Duration thresholds (in log values, mean ± SEM) as a function of IQ, for general (D), verbal (E), and non-verbal (F) intelligence. Green squares, duration thresholds for the large stimulus (D_large_); blue dots, duration thresholds for the small stimulus (D_small_). *Green lines*, fitted regression lines for general intelligence: D_large_ = 1.24 + 0.007 × IQ_g_; verbal intelligence: D_large_ = 1.32 + 0.006 × IQ_v_; and non-verbal intelligence: D_large_ = 1.64 + 0.003 × IQ_n-v_. *Blue lines*, fitted regression lines for general intelligence: D_small_ = 1.72–0.001 × IQ_g_; verbal intelligence: D_small_ = 1.59–0.0001 × IQ_v_; and non-verbal intelligence: D_small_ = 1.90–0.003 × IQ_n-v_. Pearson correlation (*r*) and *p* values are inserted in each panel. Dashed lines: 95% regression confidence interval.

Fig 4 shows the scatter plots for the motion suppression index (MSI) as a function of the IQ values for general intelligence (Fig 4A), verbal intelligence (Fig 4B), and non-verbal intelligence (Fig 4C). These results show a significant correlation between general intelligence (RIAS(IQ)) and MSI (*r* = 0.43, *p* = 0.002, 95% CI = [0.17, 0.64], N = 47). This replicates the findings from Melnick et al. (2013) (*r* = 0.71, p<0.001, N = 53), although our correlation is smaller. The screening IQ test (RIST, results not shown in the Fig 4) shows a significant positive correlation too (*r* = 0.38, *p* = 0.008, 95% CI = [0.11, 0.60], N = 47). There is also a significant correlation between Verbal(IQ) and MSI (*r* = 0.37, *p* = 0.012, 95% CI = [0.09, 0.59], N = 47) and Non-Verbal(IQ) and MSI (*r* = 0.34, *p* = 0.019, 95% CI = [0.06, 0.57], N = 47). This result is consistent with those from the study performed by Melnick et al. (2013), who found a strong correlation between Verbal Comprehension and MSI (*r* = 0.69, *p*<0.001, N = 53) too. After applying the Bonferroni correction for multiple comparisons, only the correlation Non-Verbal(IQ) vs. MSI turns out not to be significant *p* = 0.076 (*p* = 0.019 x 4). However, when the number of planned correlations is small, no correction is advised [71].

Our results show that MSI increases with increasing IQ, therefore, this means that the difference between duration thresholds for the large stimulus and the small stimulus increase with increasing IQ. Fig 4D, 4E and 4F show this. Only for Non-Verbal intelligence and for the duration thresholds for the small stimulus we found a significant negative correlation (*r* = -0.37, *p* = 0.011, 95%CI = [-0.09, -0.59], N = 47). Melnick et al. [1] found a significant negative correlation for general IQ and duration thresholds for small stimulus (*r* = -0.46, *p* = 0.0005). This suggests that participants with a higher IQ processed the motion of the small stimulus faster than participants with a lower IQ. For the large stimulus, we found positive correlations, although not significant. Our results also show that the correlations between IQ and duration thresholds for the small and large stimuli were significantly different for general intelligence (Fig 4D; RIAS (IQ), (z = 2.241, *p* = 0.025); and Non-verbal intelligence (Fig 4F; Non-verbal (IQ), z = 2.242, *p* = 0.025).

In order to control for the shared variance between the performance for the small and large stimuli, we calculated semipartial correlations between the IQ values and the duration thresholds for the aforementioned stimuli; we did this for the three IQ measurements. In the case of general intelligence (Fig 4D), we found significant negative semipartial correlations between IQ and the small stimulus (sr = -0.4; *p* = 0.005) and significant positive correlations between IQ and the large stimulus (sr = 0.42, *p* = 0.003). For verbal intelligence (Fig 4E), we only found significant a positive semipartial correlation for the large stimulus (sr = 0.36, *p* = 0.012). For non-verbal intelligence (Fig 4F), we found a significant negative semipartial correlation for the small stimulus (sr = -0.48, *p* < 0.001) and a significant semipartial correlation for the large stimulus (sr = 0.33, *p* = 0.02).

A multiple linear regression using the large and small stimuli as predictors of intelligence confirmed the opposite relationship between IQ and the duration thresholds for the small and large stimuli. When it comes to general intelligence (Fig 4D), R^2^ = 0.23, F_2,44_ = 6.41, *p* = 0.004, variance inflation factor (VIF = 1.36); duration thresholds for the small stimulus decrease with increasing IQ (using standardized data, β = -0.47, t_44_ = -3.02, *p* = 0.004); and duration thresholds for the large stimulus increase with increasing IQ (β = 0.49, t_44_ = 3.2, *p* = 0.003). For verbal intelligence (Fig 4E), R^2^ = 0.135, F_2,44_ = 3.44, *p* = 0.048; only the duration thresholds for the large stimulus increase with increasing IQ (β = 0.42, t_44_ = 2.59, *p* = 0.013). For non-verbal intelligence (Fig 4F); R^2^ = 0.241, F_2,44_ = 7, *p* = 0.002; once again, duration thresholds for the small stimulus decrease with increasing IQ (β = -0.56, t_44_ = -3.67, *p* < 0.001); and duration thresholds for the large stimulus increase with increasing IQ (β = 0.38, t_44_ = 2.49, *p* = 0.016). The analysis of the data using the screening IQ test (RIST) revealed similar results to those using general intelligence IQ (R^2^ = 0.19, F_2,44_ = 5.03, *p* = 0.01) both for the small (β = -0.44, t_44_ = -2.76, *p* = 0.008) and the large stimulus (β = 0.43, t_44_ = 2.75, *p* = 0.009).

Thus, our results from the motion experiment confirm the link between low-level sensory visual suppression and intelligence [1].

### Testing the link between IQ and Contrast Suppression Index

**Fig 5:**
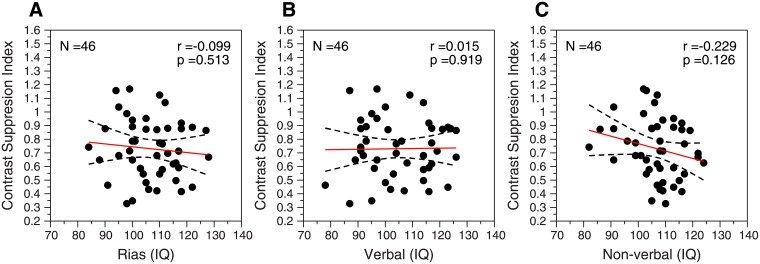
Results from the spatial experiment. Contrast suppression index (CSI) as a function of IQ for general (A), verbal (B), and non-verbal (C) intelligence. *Red lines*, fitted regression line for general intelligence: CSI = 0.95–0.002 × IQ_g_; verbal intelligence: CSI = 0.70–0.0002 × IQ_v_; and non-verbal intelligence: CSI = 1.31–0.005 × IQ_n-v_. Pearson correlation (*r*) and *p* values are inserted in each panel. Dashed lines: 95% regression confidence interval.

Fig 5 shows the results for the spatial experiment. In all cases, we found no correlation between CSI and the three IQ measurements: general intelligence (Fig 5A; *r* = -0.099, *p* = 0.513, N = 46), verbal intelligence (Fig 5B, *r* = 0.02, *p* = 0.919, N = 46), and non-verbal intelligence (Fig 5B, *r* = -0.23, *p* = 0.126, N = 46). The screening IQ test (RIST) also showed no correlation between IQ and CSI (*r* = -0.13, *p* = 0.38, N = 46). All these correlations were also computed using the no-surround condition in order to calculate the contrast suppression index (CSIns). Our results show very similar correlations (N = 46): CSIns vs. RIAS: r = -0.086, p = 0.566; CSIns vs. verbal: r = -0.004, p = 0.977; CSIns vs. non-verbal: r = -0.177, p = 0.239; CSIns vs. RIST: r = -0.154, p = 0.306.

We also computed the correlations using the absolute contrast thresholds (no-surround, orthogonal surround, and parallel surround) and all of them were not correlated. For the no-surround condition (N = 46), NS vs. RIAS: *r* = -0.047, *p* = 0.756; NS vs. verbal: *r* = 0.01, *p* = 0.945; NS vs. non-verbal: *r* = -0.142, *p* = 0.347; NS vs. RIST: *r* = 0.04, *p* = 0.787. For the orthogonal surround condition (N = 46), OS vs. RIAS: *r* = -0.027, *p* = 0.785; OS vs. verbal: *r* = -0.033, *p* = 0.827; OS vs. non-verbal: *r* = -0.04, *p* = 0.782; OS vs. RIST: *r* = -0.023, *p* = 0.879. And for parallel surround condition (N = 46), PS vs. RIAS: *r* = -0.09, *p* = 0.526; PS vs. verbal: *r* = 8.5×10^−5^, *p* = 0.999; PS vs. non-verbal: *r* = -0.213, *p* = 0.155; PS vs. RIST: *r* = -0.122, *p* = 0.416.

## Discussion

The main objective of this study was to test the link between visual surround suppression and intelligence. Previous results did find a strong link between motion surround suppression and IQ [1] and between contrast surround suppression and IQ [5]. Here we wanted to test this link using a similar motion suppression and contrast suppression task, but this time measuring contrast detection thresholds. Our results from the psychophysical experiments show the classic findings. For the motion discrimination task, we have found that at high contrasts, duration thresholds were higher for the large stimulus compared to the small one. On the other hand, for the contrast detection task, detection thresholds where higher when the target and the surround had the same orientation compared to targets and surrounds with orthogonal orientation or targets without a surround. The strength of the suppression in both tasks was quantified by a motion and a contrast suppression index. We have correlated both suppression indices and have found the correlation to be non-significant (*r* = 0.10, *p* = 0.47), thus replicating the main finding of Yazdani et al. (2015, *r* = -0.19, *p* = 0.24). These results suggest that motion and contrast surround suppression reflect the activation of independent cortical mechanisms. Although these two measurements are uncorrelated, still we could expect that both measurements would highly correlate with a third variable (IQ). For example, Cook et al. [5] showed that first and second-order surround suppression strength do not correlate, but both of them do correlate with GABA concentration.

Our results show that only the motion surround suppression index (MSI) correlates with IQ, in particular, we have found significant positive correlations between MSI and general (*r* = 0.43), verbal (*r* = 0.37), and non-verbal (*r* = 0.34) intelligence.

These results replicate previous findings, even though the correlations are weaker than in the original study (general intelligence: *r* = 0.71) [1]. The biggest difference between both studies is the intelligence test used. Melnick et al. [1] administered a short version of WAIS-III [72] in the first experiment and the full-length WAIS-IV [73] in the second one. In both experiments, they obtained similar results. Conversely, we have used the RIAS test and its screening version, the RIST. Both tests are highly correlated with WAIS-III, so probably the IQ test used is not responsible for the differences in the correlation values between ours and Melnick et al’s [1] study. The straightforward explanation for these differences could be due to the different range of IQs used in both studies. The average IQ in Melnick et al’s. [1] study is 112.92 (N = 53) and the one in our study is 106.2 (N = 47). When re-analysing the data of Melnick et al. [1], if we eliminate those participants with IQs higher than 120, then the average IQ becomes 106.4 (N = 36), a value similar to our results. Then, the correlation between IQ and MSI becomes *r* = 0.41, *p* = 0.013 (N = 36), which is similar to the one we have found. This means that the strong correlation found by Melnick et al. [1] is probably driven by participants with very high IQs. Thus, this replication shows that although it is known that people with high IQ are faster when it comes to processing visual information [74–76], the correlation between MSI and IQ cannot be explained solely by the speed of visual processing. Although our results show a significant positive correlation between the duration thresholds for small and large stimuli (*r* = 0.51), when these are correlated with IQ, we find opposite correlations. This is, a positive correlation for the large stimulus and a negative correlation for the small stimulus. Therefore, participants with a high IQ show higher MSI values because they tend to perform better in the case of the small stimulus (lower duration thresholds), and motion discrimination is impaired for the large stimulus (higher duration thresholds).

Although our results replicate Melnick et al’s results (with a weaker correlation), it is important to note that, in a recent study, Troche et al. [4] couldn’t replicate them. They found significant negative correlations between the duration thresholds for all the sizes they tested and the *g* factor. Therefore, the correlation between the suppression index (MSI) and the *g* factor (IQ) was practically 0 (*r* = -0.01, *p* = 0.84, N = 177). These authors also used a different intelligence test which was a short form of the Berlin Intelligence Structure test [77]. Previous studies have shown that different IQ tests are highly correlated [78], so we can assume that the differences between the IQ test used by Melnick et al. [1], Troche et al. [4] and our study are not related to the differences obtained in the results. The study of Troche et al. [4] presents a very high statistical power given by the large sample of participants they used (N = 177), thus it might be possible that because of the smaller sample used in our study (N = 47) and Melnick et al., [1] (N = 53), we are giving rise to false positive results. However, the results of Troche et al. [4] present incongruences when compared to previous psychophysical findings. For example, the average MSI of Troche et al. [4] study is much smaller (MSI = 0.22 ± 0.16, N = 177) than the one from Melnick et al., [1] (MSI = 0.32 ± 0.15, N = 53), Yazdani et al. [8] (MSI = 0.40 ± 0.22, N = 36), Read et al. [39] (MSI = 0.31 ± 0.16, N = 56), and the present study (MSI = 0.37 ± 0.14, N = 47). This becomes more surprising when we compare the age of the participants between the studies. In Troche el at’s. [4], the mean age was 21.1 ± 2.7 years, in Melnick et al. [1] 33.14 ± 13.36 years, in Yazdani et al’s [8] 42.3 years, and in our study it was 20.74 ± 2.44 years. There is multiple psychophysical evidence indicating that motion surround suppression (MSI) decreases with age [8, 22, 24, 25]. Therefore, one would expect that the study of Troche et al. [4] should show a much higher MSI than Melnick et al. [1]. This is even more surprising if we take into account that Troche et al. [4] used higher contrasts (95%) than Melnick et al. [1] (42%). Higher contrasts rise the MSI because they facilitate the discrimination of small stimuli (lowering the duration thresholds) and increase the strength of the surround suppression for large stimuli (increasing the duration thresholds) [9].

However, the biggest difference between the results from Troche et al., [4] and previous studies lies in the average of the duration thresholds for the smallest size tested (1.8 deg) (82 ± 28 msec, mean ± SD). Using lower contrast (42%), Melnick et al. [1] found an average of 39.13 ± 17.5 msec, and in our study, using a similar contrast (92%) to that of Troche et al. [4], we found an average of 38.2 ± 8.37 msec.

All these differences in the psychophysical results could be explained by the different equipment used in the study of Troche et al. [4]. All previous studies used CRTs or DLP projectors (Depth-Q 360, Cambridge Research Systems, UK) [1, 8, 9, 39]. Conversely, in Troche el al. [4] study they used an LCD display. We don’t know, however, whether the particular characteristics of LCD displays might be responsible for these differences. Troche et al. [4] suggested that this weaker suppression could be related to an attenuation of the onset transient provided that surround suppression is weaker for weaker transients [79]. Future studies should compare CRT monitors to LCD displays in order to find out the effect of the onset transients on motion discrimination.

Finally, our results from the spatial experiment showed no correlation between contrast surround suppression (CSI) and general (*r* = -0.09, *p* = 0.52, N = 46), verbal (*r* = 0.015, *p* = 0.919, N = 46), and non-verbal intelligence (*r* = -0.23, *p* = 0.13, N = 46). These results do not show the strong correlation between suppression index and visuospatial IQ (*r* = 0.87, *p* = 0.0021, N = 9) found by Cook et al. [5]. Although both studies measure contrast surround suppression, there are experimental differences that may explain this discrepancy. One of the differences lies in the IQ test used; unlike us, Cook et al. [5] administered the Weschler Abbreviated Scale of Intelligence (WASI, [80]), but as we stated before, different IQ test are usually highly correlated [78]. Another difference is the small number of participants used (N = 9) by Cook et al. [5]. Although this small number of participants could have led to a false positive result, the authors provide a robust measurement from the psychophysical task (they average results across different eccentricities), and also those measurements are highly correlated with GABA. It is therefore unlikely that Cook et al. [5] finding were accidental. On the other hand, one significant methodological difference between both studies is that in our experiment we performed a contrast detection task and Cook et al. [5] performed a contrast matching task. Previous psychophysical results have shown a similar behavior of the contrast surround suppression mechanism for contrast detection and contrast matching tasks. For example, for both kinds of tasks, contrast surround suppression is stronger in the periphery and weaker in the fovea [6, 45]; suppression is stronger for parallel surrounds than for orthogonal surrounds [6, 7, 45, 57, 61]; contrast surround suppression is spatial-frequency tuned [6, 7, 56, 57], and contrast surround suppression increases when increasing the contrast of the surround [6, 61]. All these similar characteristics in contrast detection and contrast matching suggest a similar mechanism underlying contrast surround suppression. However, there are also differences. For example, Xing & Heeger, [45], using a contrast matching task, found that the orientation and the spatial frequency of the surround does not have a strong impact on surround suppression in the periphery, whereas other studies that used a contrast detection task found that surround suppression in the periphery was orientation tuned (full bandwidth at half function about 30 deg) [6], and spatial-frequency tuned (between 1 and 3 octaves) [6, 7]. In Xing & Heeger, [45] and Petrov et al., [6] the authors suggest that center-surround interactions may have different functional roles in the fovea and the periphery. Recently, using a contrast matching task, McKendrick et al., [81] have found that surround suppression in the fovea, is larger in older adults, whereas Nguyen & McKendrick [82] have found the opposite in the periphery (6 degrees eccentricity). On the other hand, surround suppression in the parafovea (4–5 degrees eccentricity) (measured using contrast thresholds) remains constant between the ages of 20 and 70 years [8, 48]. These results suggest that contrast surround suppression in the fovea and in the periphery, as well as contrast surround suppression measured with thresholds or perceived contrast likely reveal independent neuronal mechanisms [83]. In our contrast suppression experiment we measured detection thresholds in the periphery (5 deg eccentricity) whereas in Cook et al. [5] they averaged their suppression indices across four eccentricities (0, 3, 6, and 9 deg). Consequently this makes our results difficult to be compared with Cook et al’s. Therefore, it could be suggested that the activation of different contrast suppression mechanisms explains the absence of a correlation in our study, but not in Cook et al’s study. However, the evidence about two different contrast suppression mechanisms for contrast thresholds and contrast matching is less compelling than the similar surround suppression properties revealed by both measurements.

## Conclusion

Our results from the motion experiment showed that in order to discriminate the correct direction of motion, participants with a higher IQ needed shorter presentation durations for the small stimulus and longer durations for the large stimulus. Therefore, the speed of processing (measured with the small stimulus) and perceptual suppression (measured with the large one) showed opposite correlations with IQ. These results support the link between motion surround suppression and IQ previously found by Melnick et al. [1].

Our results from the spatial experiment showed no correlation between contrast surround suppression measured with contrast thresholds and IQ.

## Supporting information

S1 Dataset The data presented in this paper. (XLSX)
